# Soaking RNAi in *Bombyx mori* BmN4-SID1 Cells Arrests Cell Cycle Progression

**DOI:** 10.1673/031.013.15501

**Published:** 2013-12-19

**Authors:** Hiroaki Mon, Zhiqing Li, Isao Kobayashi, Shuichiro Tomita, JaeMan Lee, Hideki Sezutsu, Toshiki Tamura, Takahiro Kusakabe

**Affiliations:** 1 Laboratory of Silkworm Science, Kyushu University Graduate School of Bioresource and Bioenvironmental Sciences, Hakozaki 6-10-1, Fukuoka 812-8581; 2 Transgenic Silkworm Research Center, National Institute of Agrobiological Sciences, 1-2 Owashi, Tsukuba, Ibaraki 305-8634, Japan

**Keywords:** RNAi, SID-1, dsRNA, silkworm

## Abstract

RNA interference (RNAi) is an evolutionarily conserved mechanism for sequence-specific gene silencing. Previously, the BmN4-SID1 cell expressing *Caenorhabditis ele gans* SID-1 was established, in which soaking RNAi could induce effective gene silencing. To establish its utility, 6 cell cycle progression related cDNAs, *CDK1, MYC, MYB, RNRS, CDT1*, and *GEMININ,* were isolated from the silkworm, *Bombyx mori* L. (Lepidoptera: Bombycidae), and their expressions were further silenced by soaking RNAi in the BmN4-SID1 cells. The cell cycle progression analysis using flow cytometer demonstrated that the small amount of double stranded RNA was enough to arrest cell cycle progression at the specific cell phases. These data suggest that RNAi in the BmN4-SID1 cells can be used as a powerful tool for loss-of-function analysis of *B. mori* genes.

## Introduction

The silkworm, *Bombyx mori* L. (Lepidoptera: Bombycidae), is one of the most important model insects for Lepidoptera, which includes the most highly destructive agricultural pests. Recently, *B. mori* larvae and pupae were used as an insect factory system for the large-scale production of useful recombinant proteins (Lee et al. 2012). Due to these agricultural and industrial applications, the establishment of a convenient and effective method for gene function analysis is needed in this insect.

RNA interference (RNAi) is a conserved gene silencing mechanism triggered by double stranded RNA (dsRNA). RNAi knockdown experiments have been successfully performed in cultured *B. mori* cells by transfecting dsRNAs or expressing hairpin RNAs (Tsukioka et al. 2006; Fujita et al. 2009; Terenius et al. 2011). The cytotoxicity and low efficiency of transfection, however, restrict its application for experiments requiring the “whole” cell population. In contrast to mammalian cells, non-sequence specific suppression of gene expression in response to long dsRNA was not observed in insect cells, including *B. mori* cells (Sledz et al. 2003). In *Drosophila melanogaster* Meigen (Diptera: Drosophilidae) S2 cells, long dsRNA is rapidly bound on the cell surface and autonomously taken into the cells (Saleh et al. 2006). Therefore, soaking RNAi would be an ideal method to induce specific gene silencing in *B. mori* cells without activating undesirable PKR/RNaseL pathways (Sledz et al. 2003).

Recently, we reported the construction of the BmN4-SID1 cell lines ectopically expressing *Caenorhabditis elegans* transmembrane protein SID-1, which functions as a channel for the transport of dsRNA (Winston et al. 2002). The expression of *Caenorhabditis elegans* transmembrane protein SID-1 could trigger effective gene silencing in the BmN4-SID1 cells without affecting the cell viability. Moreover, high-throughput RNAi screenings have become a widely used method in model organisms (Mohr et al. 2010).

In the present study, regulation of cell cycle progression was chosen as a model mechanism to further explore RNAi efficiency in the BmN4-SID1 cells. Six *B. mori* cDNAs, *CDK1, MYC, MYB, RNRS, CDT1*, and *GEMININ,* were cloned, and the effects of their knockdown upon cell cycle progression were analyzed. These data demonstrated the conspicuous usability of the BmN4-SID1 cells, and high-throughput RNAi screenings using this cell line will become a widely used approach for gene function analysis in *B. mori.*

## Materials and Methods

### Cell culture

The BmN4-SID1 cells constructed from BmN4 cells (from Dr. Chisa Aoki, Kyushu University Graduate School) were cultured in IPL-41 medium (Sigma-Aldrich, www.sigmaaldrich.com) supplemented with 10% fetal bovine serum (Mon et al. 2012). In the BmN4-SID1 cells, *Caenorhabditis elegans* transmembrane protein SID-1 mRNA was overexpressed under the control of a strong viral OpIE2 promoter (Invitrogen, www.invitrogen.com).

### RT-PCR

Semi-quantitative reverse transcription polymerase chain reaction (RT-PCR) was performed as described by Mon et al. (2004) and Tsukioka et al. (2006), except for the primers used. The primers used for RT-PCR in our study are listed in Table 1.

**Table 1.**
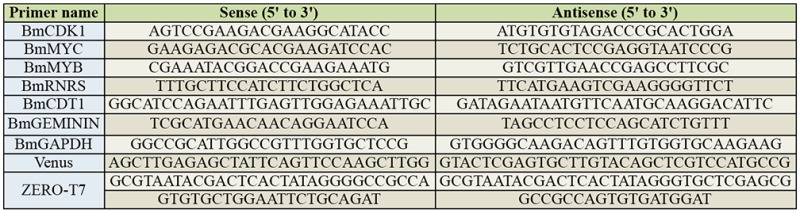
List of primers used in this study.

### RNAi

Double-stranded RNA was transcribed in vitro using T7 RNA polymerase as described by Tsukioka et al. (2006). The DNA fragments containing partial cDNA sequences for *CDK1, MYC, MYB, RNRS, CDT1, GEMININ,* and a *GFP* variant gene *(Venus)* were amplified by PCR using the primers listed in Table 1. The PCR products were cloned into an EcoRV site of pZErO-2 (Life Technologies, www.lifetechnologies.com). The T7 promoter sequences were added on both termini of the target DNA fragments by PCR using ZERO-T7 primers (Table 1). The fragments with 2 T7 promoter sequences were transcribed by T7 RNA polymerase. To induce RNAi in BmN4-SID1 cells, dsRNAs were added to the IPL-41 medium directly.

### Flow cytometry

Flow cytometry analysis was performed with a Guava PCA-96 Flow Cytometer (Millipore, www.millipore.com) and the obtained data was analyzed using FlowJo software (Tree Star, www.treestar.com). Cells were fixed by adding 70% ethanol and kept at 4° C until used. Fixed cells were washed with PBS and then treated with RNaseA. Cells were stained by propidium iodide and analyzed immediately by the flow cytometer.

## Results and Discussion

### Identification and expression profiles of the *Bombyx mori* cell cycle progression related genes

By RNAi screening of 11,971 *D. melanogaster* genes, Bjorklund et al. (2006) found that depletions of 270 and 169 genes resulted in significant changes in G1 and G2 populations, respectively (Bjorklund et al. 2006). From these, 6 genes exhibiting strong and typical RNAi phenotypes were selected as targets. The nucleotide sequences for *D. melanogaster CDK1, MYC, MYB, RNRS, CDT1,* and *GEMININ* were downloaded from NCBI (http://www.ncbi.nlm.nih.gov/). Using these sequences as queries, a NCBI TBLASTN search against the updated *B. mori* genome sequence was performed, and a full-length cDNA sequence for each *B. mori* ortholog was assembled. The 6 *B. mori* genes determined in this study were deposited in Gen-GenBank under the accession numbers of NM_001044047, AB649263, AB703262, AB703263, AB703260, and AB703261, respectively.

**Figure 1.**
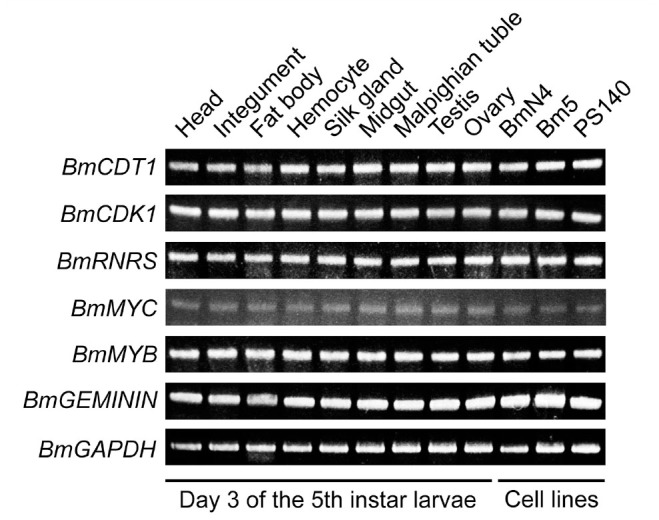
Tissue and cell line-specific expression patterns of *Bombyx mori* cell cycle progression related genes. RT-PCR was performed to detect the expression patterns of 6 *B. mori* genes using the specific primers, and *BmGAPDH* gene was used as the internal control. High quality figures are available online.

To examine the expression pattern of the 6 *B. mori* genes identified, semi-quantitative RT-PCR analysis was performed in various tissues from larvae on day 3 of the fifth instar and several *B. mori* cell lines. As expected, all cell cycle progression related genes were expressed ubiquitously (Figure 1).

### Knockdown of G1 cell cycle regulators, *BmCDT1* or *BmMYC*, expression by soaking RNAi

Like the *D. melanogaster* S2 cells, the stably transformed BmN4-SID1 cells uptake dsRNA spontaneously (Saleh et al. 2006; Bettencourt-Dias et al. 2009; Kobayashi et al. 2012; Mon et al. 2012). The results of Mon et al. (2012) implyed that the phenotype observed in the BmN4-SID1 cells by soaking RNAi was sequence-specific suppression, not an off-target effect.

**Figure 2.**
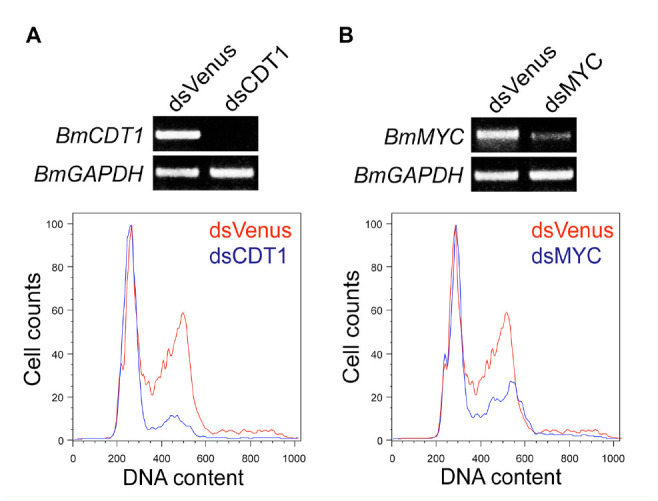
Knockdown of *BmCDT1* or *BmMYC* expression by soaking RNAi. Knockdown efficiency and cell cycle analysis for the *BmCDT I* (A) or *BmMYC* (B) depleted BmN4-SID 1 cells was performed by RT-PCR and flow cytometry, respectively. In flow cytometry analysis, the number of control cells (dsVenus treatment) is indicated by the red line, and the number of cells treated with dsRNA for the *Bombyx mori* gene is indicated by the blue line. High quality figures are available online.

To confirm utility of the BmN4-SID1 cells, soaking RNAi in *BmCDT1* or *BmMYC* was performed. Cdt1 is essential for loading Mem proteins into pre-replicative complexes during replication licensing, whereas Myc transcription factor is a key regulator of the G1 phase cell cycle. In human T lymphocytes, down-regulated c-MYC expression inhibits entry into S phase but not progress from GO to G1 (Heikkilä et al. 1987). The efficacy RNAi against 2 genes can be analyzed by flow cytometry 7 days after the soaking. As shown in Figure 2, 200 ng/mL of the dsRNAs specific for *BmCDT1 or BmMYC* arrested cell cycle progression at the G1 phase. The G1-S transition was profoundly affected by the depletion of *BmCDT1* because of its essential role in replication licensing, as 78.6% of cells were stopped at the G1 phase.

**Figure 3.**
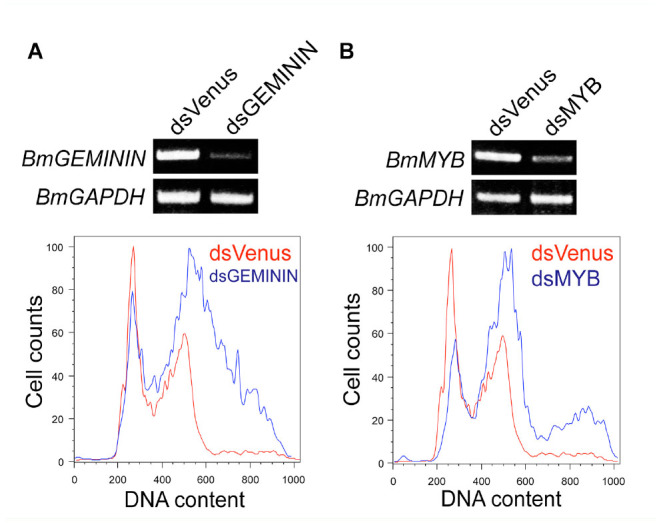
Knockdown of *BmGEMININ* or *BmMYB* expression by soaking RNAi.. Knockdown efficiency and cell cycle analysis for the *BmGEMININ* (A) or *BmMYB* (B) depleted BmN4-SID1 cells was performed by RT-PCR and flow cytometry, respectively. In flow cytometry analysis, the number of control cells (dsVenus treatment) is indicated by the red line, and the number of cells treated with dsRNA for the *Bombyx mori* gene is indicated by the blue line. High quality figures are available online.

### Knockdown of *BmGEMININ* or *BmMYB* **expression by soaking RNAi induces DNA** re-replication or DNA replication without cytokinesis

Geminin is reported to inhibit relicensing of replication by binding to Cdt1 and consequently preventing the loading of the Mcm proteins onto the pre-replication complex (Wohlschlegel et al. 2000; Tada et al. 2001). In *D. melanogaster,* the *GEMININ* depletion results in re-replication of chromosomal DNA (Mihaylov et al. 2002). Similarly, knockdown of *BmGEMININ* expression induced DNA rereplication (Figure 3). This result demonstrated that the depletion of *BmGEMININ* was sufficient to allow re-replication in *B. mori.*

Myb was first identified as a key transcription factor in the regulation of the cell cycle, and subsequently was reported to be involved in the maintenance of genome stability through the regulation of genes related to G2/M transition (Sala 2005; Lorvellec et al. 2010). In murine embryonic stem cells, knockdown of *B-Myb* results in delayed transit through the G2/M phase, severe mitotic spindle, and centrosome defects (Tarasov et al. 2008). As shown in Figure 3, the depletion of *BmMYB* arrested cell cycle progression at the G2/M phase and resulted in replication without cytokinesis. Compared to the murine ES and *D. melanogaster* S2 cells, however, the polyploid population was significantly higher in the BmN4-SID1 cells. It seems to be very easy to undergo mitosis without cytokinesis in arrested *B. mori* cells at the G2/M phase.

### Knockdown of *BmCDKl* expression by soaking RNAi arrests cell cycle progression at the G2/M phase

**Figure 4.**
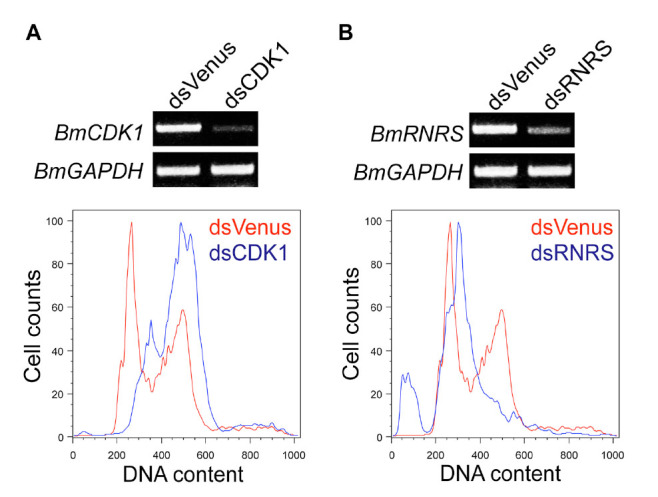
Knockdown of *BmCDK1* or *BmRNRS* expression by soaking RNAi. Knockdown efficiency and cell cycle analysis for the *BmCDK1* (A) or *BmRNRS* (B) depleted BmN4-SID1 cells was performed by RT-PCR and flow cytometry, respectively. In flow cytometry analysis, the number of control cells (dsVenus treatment) is indicated by the red line, and the number of cells treated with dsRNA for the *Bombyx mori* gene is indicated by the blue line. High quality figures are available online.

CDK1 is a Ser/Thr kinase that plays an important role in the G2/M transition process (Morgan 1995). After *BmCDKl* depletion, cell populations were increased at the G2/M phase compared to the control (Figure 4). Unlike *D. melanogaster,* in which the *CDK1* knockdown caused cell death and also a giant cell and re-replication phenotype (Bjorklund et al. 2006), the *BmCDKl* depletion failed to induce such a phenotype. In *B. mori,* Y-ray irradiation resulted in marked cells arrest at the G2/M phase but not undergoing apoptosis (Takahashi et al. 2006). Taken together, the results suggest that *B. mori* lacks death programs for apoptosis in the cells arrested at the G2/M phase.

### Knockdown of *BmRNRS* expression by soaking RNAi arrests cell cycle progression at the S phase and induces apoptosis

*BmRNRS* was depleted in the BmN4-SID1 cells. RnrS catalyzes the conversion of nucleotides to deoxynucleotides (Reichard 1993). Due to its essential role in DNA biosynthesis, the depletion of *RNRS* leads *D. melanogaster* S2 cells to S phase arrest (Bjorklund et al. 2006). In the BmN4-SID1 cells, depletion of *BmRNRS* by RNAi arrested cell cycle at the S phase (Figure 4). Unlike *D. melanogaster* S2 cells, the RNAi for *BmRNRS* was able to induce apoptosis. Interestingly, in contrast to the cells arrested at the G2/M phase, the BmN4 cells retain the ability to undergo apoptosis in response to nucleotide starvation. BmN4 cells exposed to UV irradiation temporarily arrested their cell cycle at the S phase then exited from the checkpoint, indicating that *B. mori* cells are more resistant to UV irradiation than *Spodoptera friigiperada* Sf21 cell (Takahashi et al. 2006). A possible explanation for this difference is that the cell cycle arrest caused by nucleotide starvation occurs a little earlier than that induced by UV irradiation, and *B. mori* cells might activate the cell death pathway in a narrow time window.

In conclusion, the present data demonstrated that soaking RNAi in BmN4-SID1 cells is effective in high-throughput loss-of-function analysis of *B. mori* genes.

## Abbreviations:

dsRNA,: double stranded RNA;
RNAi,: RNA interference;
RT-PCR,: reverse transcription polymerase chain reaction

## References

[bibr01] Bettencourt-Dias M, Goshima G. (2009). RNAi in *Drosophila* S2 cells as a tool for studying cell cycle progression.. *Methods in Molecular Biology*.

[bibr02] Björklund M, Taipale M, Varjosalo M, Saharinen J, Lahdenpera J, Taipale J. (2006). Identification of pathways regulating cell size and cell-cycle progression by RNAi.. *Nature*.

[bibr03] Fujita K, Sagisaka A, Tomimoto K, Ishibashi J, Imanishi S, Yamakawa M, Tanaka H. (2009). DNA vector-based RNA interference in cell lines derived from *Bombyx mori*.. *Bioscience Biotechnology and Biochemistry*.

[bibr04] Heikkilä R, Schwab G, Wickstrom E, Loke SL, Pluznik DH, Watt R, Neckers LM. (1987). A c-myc antisense oligodeoxynucleotide inhibits entry into S phase but not progress from G0 to G1.. *Nature*.

[bibr05] Kobayashi I, Tsukioka H, Kômoto N, Uchino K, Sezutsu H, Tamura T, Kusakabe T, Tomita S. (2012). SID-1 protein of *Caenorhabditis elegans* mediates uptake of dsRNA into *Bombyx* cells.. *Insect Biochemistry and Molecular Biology*.

[bibr06] Lee JM, Mon H, Banno T, Iiyama K, Kusakabe T. (2012). *Bombyx mori* strains useful for efficient recombinant protein production using a baculovirus vector.. *Journal of Biotechnology and Biomaterials*.

[bibr07] Lorvellec M, Dumon S, Maya-Mendoza A, Jackson D, Frampton J, Garcia P. (2010). B-Myb is critical for proper DNA duplication during an unperturbed S phase in mouse embryonic stem cells.. *Stem Cell*.

[bibr08] Mihaylov IS, Kondo T, Jones L, Ryzhikov S, Tanaka J, Zheng J, Higa LA, Minamino N, Cooley L, Zhang H. (2002). Control of DNA Replication and Chromosome Ploidy by Geminin and Cyclin A.. *Molecular and Cellular Biology*.

[bibr09] Mohr S, Bakal C, PerrimonN (2010). Genomic screening with RNAi: results and challenges.. *Annual Review of Biochemistry*.

[bibr10] Mon H, Kusakabe T, Lee JM, Kawaguch Y, Koga K. (2004). In vivo DNA double-strand breaks enhance gene targeting in cultured silkworm cells.. *Comparative Biochemistry and Physiology Part B*.

[bibr11] Mon H, Kobayashi I, Ohkubo S, Tomita S, Lee JM, Sezutsu H, Tamura T, Kusakabe T. (2012). Effective RNA interference in cultured silkworm cells mediated by overexpression of *Caenorhabditis elegans* SID-1.. *RNA Biology*.

[bibr12] Morgan DO. (1995). Principles of CDK regulation.. *Nature*.

[bibr13] Reichard P. (1993). From RNA to DNA, why so many ribonucleotide reductases?. *Science*.

[bibr14] Saleh MC, van Rij RP, Hekele A, Gillis A, Foley E, O'Farrell PH, Andino R. (2006). The endocytic pathway mediates cell entry of dsRNA to induce RNAi silencing.. *Nature Cell Biology*.

[bibr15] Sledz CA, Holko M, de Veer MJ, Silverman RH, Williams BR. (2003). Activation of the interferon system by short-interfering RNAs.. *Nature Cell Biology*.

[bibr16] Tada S, Li A, Maiorano D, Méchali M, Blow JJ. (2001). Repression of origin assembly in metaphase depends on inhibition of RLF-B/Cdt1 by geminin.. *Nature Cell Biology*.

[bibr17] Takahashi M, Lee JM, Mon H, Kawaguchi Y, Koga K, Kusakabe T. (2006). Cell Cycle Arrest induced by Radiation in Cultured Silkworm Cells.. *Journal of Insect Biotechnology and Sericology*.

[bibr18] Tarasov KV, Tarasova YS, Tam WL, Riordon DR, Elliott ST, Kania G, Li JM, Yamanaka S, Crider DG, Testa G, Li RA, Lim B, Stewart CL, Liu Y, Van Eyk JE, RP Wersto, AM Wobus, Boheler KR. (2008). B-MYB is essential for normal cell cycle progression and chromosomal stability of embryonic stem cells.. *PLoS One*.

[bibr19] Terenius O, Papanicolaou A, Garbutt JS, Eleftherianos I, Huvenne H, Kanginakudru S (2011). RNA interference in Lepidoptera: an overview of successful and unsuccessful studies and implications for experimental design.. *Journal of Insect Physiology*.

[bibr20] Tsukioka H, Takahashi M, Mon H, Okano K, Mita K, Shimada T, Lee JM, Kawaguchi Y, Koga K, Kusakabe T. (2006). Role of the silkworm argonaute2 homolog gene in double-strand break repair of extrachromosomal DNA.. *Nucleic Acids Research*.

[bibr21] Sala A. (2005). B-MYB, a transcription factor implicated in regulating cell cycle, apoptosis and cancer.. *Europian Journal of Cancer*.

[bibr22] Winston WM, Molodowitch C, Hunter CP. (2002). Systemic RNAi in *C. elegans* requires the putative transmembrane protein SID-1.. *Science*.

[bibr23] Wohlschlegel JA, Dwyer BT, Dhar SK, Cvetic C, Walter JC, Dutta A. (2000). Inhibition of eukaryotic DNA replication by geminin binding to Cdt1.. *Science*.

